# The Relation Between Empathy and Insight in Psychiatric Disorders: Phenomenological, Etiological, and Neuro-Functional Mechanisms

**DOI:** 10.3389/fpsyt.2019.00966

**Published:** 2020-02-06

**Authors:** Bérangère Thirioux, Ghina Harika-Germaneau, Nicolas Langbour, Nematollah Jaafari

**Affiliations:** ^1^Unité de Recherche Clinique Intersectorielle en Psychiatrie à vocation régionale Pierre Deniker, Centre Hospitalier Henri Laborit, Poitiers, France; ^2^Université de Poitiers, CHU de Poitiers, INSERM U 1084, Experimental and Clinical Neuroscience Laboratory, Groupement de Recherche CNRS 3557, Poitiers, France

**Keywords:** insight, empathy, perspective-taking, self-reflexion, psychiatric disorders, process of objectification, process of subjectification

## Abstract

Lack of insight, i.e., unawareness of one’s mental illness, is frequently encountered in psychiatric conditions. Insight is the capacity to recognize (psychical insight) and accept one’s mental illness (emotional insight). Insight growth necessitates developing an objective perspective on one’s subjective pathological experiences. Therefore, insight has been posited to require undamaged self-reflexion and cognitive perspective-taking capacities. These enable patients to look objectively at themselves from the imagined perspective of someone else. Preserved theory-of-mind performances have been reported to positively impact insight in psychosis. However, some patients with schizophrenia or obsessive-compulsive disorders, although recognizing their mental disease, are still not convinced of this and do not accept it. Hence, perspective-taking explains psychical insight (recognition) but not emotional insight (acceptance). Here, we propose a new conceptual model. We hypothesize that insight growth relies upon the association of intact self-reflexion and empathic capacities. Empathy (feeling into someone else) integrates heterocentered visuo-spatial perspective (feeling *into*), embodiment, affective (*feeling* into) and cognitive processes, leading to internally experience the other’s thought. We posit that this subjective experience enables to better understand the other’s thought about oneself and to affectively adhere to this. We propose that the process of objectification, resulting from empathic heterocentered, embodiment, and cognitive processes, generates an objective viewpoint on oneself. It enables to recognize one’s mental illness and positively impacts psychical insight. The process of subjectification, resulting from empathic affective processes, enables to accept one’s illness and positively impacts emotional insight. That is, affectively experiencing the thought of another person about oneself reinforces the adhesion of the emotional system to the objective recognition of the disease. Applying our model to different psychiatric disorders, we predict that the negative effect of impaired self-reflexion and empathic capacities on insight is a transnosographic state and that endophenotypical differences modulate this common state, determining a psychiatric disease as specific.

## Introduction

Unawareness of illness is frequently encountered in neurological [e.g., stroke (1, 2), traumatic brain injury (3), fronto-temporal dementia, Alzheimer disease (4)] and psychiatric conditions [schizophrenia (5), bipolar disorder (BD) (6–8), obsessive-compulsive disorders (OCD) (9), substance use disorders (SUD), behavioral addiction (10, 11)].

It significantly correlates with poor recovery over time and moderate to low outcomes from therapies and rehabilitation programs (12–15). Conversely, awareness of illness is associated with a better compliance to treatment, a greater efficiency of the therapeutic strategies, positive effects on prognosis, and a global amelioration of the patient’s quality of life (15–17).

The present contribution focuses on the psychiatric form of awareness of illness, which is termed “insight”, and on its deficits (18–23) (**Box 1**). Lack of insight is defined as the incapability of psychiatric patients to recognize and accept that they are suffering from a mental illness. Patients are unable to relabel their own mental events as abnormal, to identify the consequences of the illness (at physical, cognitive, and social levels), and to consent to treatment and hospitalization (19, 23–25).

Box 1 **Insight and related concepts.**

**Insight:** Psychiatric form of awareness of illness. It refers to the capability of psychiatric patients to recognize and accept that they are suffering from a mental illness. Insight is composed of three main dimensions, i.e., psychical, somaesthetic, and emotional.

**Psychical Insight:** One of the three main dimensions of insight. The psychical insight is further composed of three sub-dimensions, i.e., clinical, cognitive, and metacognitive. The psychical insight is necessary for the recognition of the mental illness.

**Clinical Insight:** One of the three sub-dimensions of the psychical insight. It refers to the awareness of symptoms and their consequences, the capacity to relabel mental events as pathological, to attribute a cause to the symptoms, and to agree with others (physicians, family, or friends) on the reality of the disease.

**Cognitive Insight:** One of the three sub-dimensions of the psychical insight. It refers to the patient’s capacity to recognize that his/her experienced cognitive deficits (e.g., attentional disorders in unipolar depression) are induced by the mental illness.

**Metacognitive Insight:** One of the three sub-dimensions of the psychical insight. It refers to the reflexive awareness of oneself as a “diseased subject”. It relies upon the capacity to make “the self as a diseased subject” a consistent object of thought.

**Somaesthetic Insight:** One of the three main dimensions of insight. Based on the awareness and representation of the bodily state, it refers to the patient’s capacity to understand that his/her physical deficits (motor, sensory, somatosensory, etc.) are an effect of the mental illness (e.g., motor slowdown in unipolar depression).

**Emotional Insight:** One of the three main dimensions of insight. It refers to the patient’s capacity of being convinced that he/she is suffering from a mental illness. The emotional insight is necessary for the acceptance of the mental illness.

**Insight growth:** Refers to the development of insight. Insight growth is specifically needed when psychiatric patients lack insight. It necessitates intact self-reflexion and empathic capacities.

**Self:** Entity which immediately and indubitably feels and knows to be the same person (ipseity) across time (temporality) and space (spatiality), to be the author of one’s own thoughts and actions (agency), and to be localized within one’s bodily borders at a given position in space (self-location).

**Self-reflexion:** Cognitive process by which an individual reasons about him/herself and evaluates whether a given attribution pertains or not to the self. It results in representations of one’s own traits, abilities, and attitudes.

Firstly, this definition indicates that there are two main stages in insight: recognition (psychical insight) and acceptance of the mental illness (emotional insight). Secondly, it suggests that insight growth necessitates the patients’ capability to develop an *objective* perspective on their *subjective* pathological experiences (**Box 1**). Insight has been, thus, posited to rely upon a combination of intact self-reflection and cognitive perspective-taking capacities (18–21, 26). These enable respectively to focus on oneself and to shift perspective. In other words, insight requires “the cognitive capacity to adopt the other’s perspective which, if intact, contributes to the metacognitive capacity to reflect upon “one’s own” mental health from the other’s perspective” (26).

Empirical studies using theory-of-mind (ToM) tasks tend to validate this assumption (27–29) (**Box 2**). It has been shown that difficulties in first-person perspective (1PP-PT) ToM tasks (with instructions neither directly evoking mental state inferences nor prompting to recruit inferential processing) in patients with schizophrenia have a more negative impact on insight, in comparison to difficulties in third-person perspective (3PP-PT) ToM tasks (with instructions directly asking what *X* thinks) (26). Hence, insight is better predicted by performances to tasks in which a more spontaneous processing is calculated. Langdon and Ward (26) concluded that theoretically reasoning about the other’s thought without simulating his/her perspective is not sufficient for insight growth.

Box 2 **Empathy and related concepts.**

**Empathy:** Critical social skill. It refers to the capacity to feel and understand the lived experiences of someone else while mentally adopting his/her visuo-spatial and psychological perspective and maintaining self-other distinction. It recruits embodiment, heterocentered visuo-spatial perspective-taking, emotional/affective, higher-order cognitive, and self-regulatory processes. Under specific conditions, empathy further motivates concern and helping behavior.

**Embodiment:** One of the core processes of empathy. This automatic process enables individuals to directly and internally reproduce what another person is currently experiencing, regardless of the experiential content (motor, somatosensory, emotional, intentional, etc.).

**Spatial and temporal decentering:** Mental process enabling individuals to disengage themselves from here and now, i.e. from their own lived experience. It is required for visuo-spatial perspective-taking in empathy.

**Perspective-taking:** General term referring to a process by which individuals mentally adopt the perspective of someone else. There are different levels of perspective-taking: visual, visuo-spatial, or cognitive.

*Visual perspective-taking* enables to understand what people can see from their visual perspective (simple visual perspective-taking) and how people can see things differently (complex visual perspective-taking).

*Heterocentered visuo-spatial perspective-taking* enables individuals to mentally locate themselves into the body of someone else and to experience the world from his/her body position in space (heterocentered). It relies upon specific body-related mental imagery and transformation. Heterocentered visuo-spatial perspective-taking is one of the core processes of empathy and is specific to empathy

*Cognitive perspective-taking* is used in ToM and enables to understand the psychological perspective and mental state of someone else. According to the theory-theory, complex and higher-order cognitive perspective-taking uses logical inferences and contextual information to understand the other’s mental state, i.e., “from the outside looking in”. It is termed “third-person perspective-taking” (3PP-PT) (26). According to the simulation theory, individuals mentally put themselves in the other’s shoes and use simulation processes to understand the other’s mental state, i.e., “from the inside looking out” (26). It is termed “first-person perspective-taking” (1PP-PT).

**Theory-of-mind (ToM):** Cognitive ability enabling to represent and understand the mental states of others, including their emotions. According to the *theory-theory* account of ToM, individuals develop already in their childhood a theory about the mind of others. It enables them to reason about the thoughts and mental states of someone else with the help of logical inferences and contextual information.

According to the *simulation theory* account of ToM, individuals adopt the psychological perspective of another person while mentally putting themselves in the other’s shoes and using simulation processes.

C*ognitive ToM-like processes* are core processes of empathy. They enable individuals to understand the other’s current mental state and representations and how these may be similar to or different from their own representations.

**Self-regulation:** One of the core processes of empathy. This inhibitory process plays an important role for self-other distinction. At the *emotional level*, it enables to inhibit negative emotions that are spontaneously triggered when individuals are confronted with the other’s distress. At the *visuo-spatial level*, it enables to partially inhibit one’s egocentered visuo-spatial perspective. It is central for a balanced ego-heterocentered referential coding. At the *cognitive level*, it enables to decouple representational mechanisms associated with one’s and other’s mental state. Self-regulation also monitors the tendency to project onto someone else what individuals have already felt in similar past situations or would have potentially felt.

**Metacognition:** Cognitive ability that refers to the knowledge that individuals have about (1) their own cognitive processes, constructs, and productions (the content of the mental act), (2) their own mental states, intentions, beliefs, emotions, etc. (the form of the mental act), and (3) their capacity to analyze, understand, and think about their thoughts.

**Simulation:** Automatic process based on imagination that enables to mentally experience the others’ mental states, including their emotions. It is specifically used in first-person perspective-taking (1PP-PT). When simulating, individuals mentally use and project onto someone else their own perceptive, emotional, and cognitive schemas and patterns. It potentially leads to egocentric biases.

**Emotional contagion:** Automatic process by which an individual (*A*) attributes to himself/herself the emotion of another individual (*B*). It leads to a self-other confusion in which *A* is experiencing the same emotional state as *B* although this emotion originally stems from *B* and does not directly concern *A*. Emotional contagion is the hallmark of sympathy in contrast to empathy.

However, it has been reported that certain patients with schizophrenia (30) or OCD (31) recognize their mental illness but are not convinced of this. These data suggest that 1PP-PT ToM capacities, even in association with undamaged self-reflexion abilities, are not sufficient for the acceptance of the mental illness. Accordingly, perspective-taking only partly explains insight: that is, psychical insight (recognition) but not emotional insight (30) (acceptance) (**Box 1**).

To overcome these limitations, we propose a new conceptual model. It aims to explain the dysfunctional mechanisms underpinning lack of insight in psychiatric disorders. On the basis of empirical data showing that empathizing with someone else at both the affective and cognitive levels increases insight in schizophrenic patients (32), we hypothesize that insight growth (**Box 1**) relies upon the relationship between intact self-reflexion and empathic capacities.

Empathy (feeling into someone else) integrates heterocentered visuo-spatial perspective-taking (feeling *into*), embodiment, emotional/affective processes (*feeling* into), and higher-order cognitive representations (33–36) (**Box 2**). It enables to internally and more efficiently experience the other’s thoughts, beliefs, emotions, etc. We posit that this subjective experience (based on feelings) enables to better understand the other’s thought about oneself and facilitates the affective adherence to this thought.

Firstly, we hypothesize that empathic heterocentered, embodiment and cognitive processes, in association with self-reflexion capacities, result in the “process of objectification” (37). Generating an objective viewpoint on oneself, it enables to recognize one’s own illness. The process of objectification positively impacts psychical insight.

Secondly, we hypothesize that empathic affective processes, in association with self-reflexion capacities, result in the “process of subjectification”. That is, affectively experiencing the thought of another person about oneself reinforces the adherence of the emotional system to the objective evaluation and recognition of the illness. The process of subjectification enables to accept one’s mental illness and positively impacts emotional insight.

We further apply our conceptual model to different psychiatric disorders: schizophrenia, unipolar, and bipolar mood disorders and OCD. (1) We propose that the negative effect of impaired self-reflexion and empathic capacities on insight is a transnosographic state. (2) We predict how endophenotypical differences modulate this common state, determining a psychiatric disease as specific.

## Phenomenology of Insight

Assessment of insight is consensually admitted as standard to the examination of the patients’ general mental state and fundamental for diagnosis in psychiatry (23). However, there are still important debated issues. What etiopathogenic model does at best explain lack of insight? What is the phenomenal nature of insight?

### Different Etiopathogenic Models of Lack of Insight

Five etiopathogenic models may be distinguished [for a review, see (15); see also (22, 26, 27)]. Because our working hypotheses are based upon the continuous model, this one is described in more details in a separate subdivision.

#### The Clinical, Psychological, Neuropsychological, and Neuro-Anatomical Models

##### The Clinical Model

The clinical or categorical model assumes that lack of insight is an isolated and constitutive symptom of the disease [(38); see (27)]. Lack of insight is defined as a stable trait, i.e., as a symptom being either present or absent [“all-or-none approaches”; for a criticism, see (22)]. This model specifically aims to account for lack of insight in psychoses. It posits that psychotic patients are not able to recognize their own illness. Here, the process of recognition is first impaired and impacts, as a consequence, the process of acceptance. In clinical studies using categorical methodologies, 70% of patients with schizophrenia were found to have a low insight [for a criticism, see (39, 40)] whereas OCD, as an example, was associated with a good insight [for a criticism, see (41)]. However, experimental data only little endorse the clinical model. It is probably due to that this model hardly produces testable hypotheses (15).

##### The Psychological Model

According to the psychological model, lack of insight is caused by a psychological defense mechanism. This form of denial is a copying strategy enabling to face up to the experienced stressful events [i.e., the illness itself; (15)] and to avoid distress and the negative consequences of a diminished self-esteem [(42, 43); see (15, 26, 27)]. Here, patients recognize their own illness but do not accept it. For instance, in first-episode schizophrenia, non-stabilized compared to stabilized patients exhibited defense mechanism in a more pronounced way (N = 52). This defensive attitude was further associated with a more severe lack of insight (43) [see also (28, 44, 45)]. Although interesting, this model does not disentangle whether defense mechanism is the primary cause of lack of insight or a secondary effect of the stigmatization that is often associated with psychiatric diseases (26).

##### The Neuropsychological Model

The neuropsychological model assumes that lack of insight originates in neuro-functional abnormalities and associated impairments of neuropsychological functions (e.g., memory, executive functions, etc.). Experimental results are still contradictory. Some studies report that better insight positively correlates with higher levels of intelligence [e.g., (12, 46)], greater executive functions, and augmented frontal activity in schizophrenia [e.g., (43, 47–49) (meta-analysis on 35 studies)]. Other studies failed to find this association [(50, 51) (N = 75)]. According to Langdon and Ward (26), it means that the deficits measured by standardized neuropsychological tests are not sufficient to account for lack of insight in most of its phenomenal and clinical manifestations. Another possible explanation is that the findings are dependent upon the evaluation timing. First-episode onset, decompensation, stabilization, or remission phases may have an important impact on the association between insight and neuropsychological functions (15). This further tends to validate the continuous model.

##### The Neuro-Anatomical Model

According to the neuro-anatomical model, insight deficits correlate with reduced cerebral volume in the dorso-lateral prefontal cortex (dlPFC), medial PFC (mPFC), anterior and posterior cingulate cortex (ACC/PCC), insula, temporo-parietal junction (TPJ), and hippocampus [(52) (N = 15); (53, 54) (N = 35); (55) (N = 14); (56–60)]. Neuroimaging data do not reach consensus. Again, this is probably due to that sample sizes are often small, leading to biased statistical analyses (15).

Although importantly contributing to the exploration of lack of insight, these four models have not been validated by consistent empirical data so far. In addition to methodological shortcomings (sample size, statistical methods, standardized test batteries, testable hypotheses, etc.), these contradictory results could be further due to the timing of evaluation (acute episode, remission phase, etc.). If correct, it suggests that insight may be variable over time and in intensity, depending upon multiple factors. This observation is the starting point of the continuous model.

#### The Continuous Model

The continuous model evidenced the complexity of insight. It does not define insight as a stable symptom of the disease but “a continuum of thinking and feeling, affected by numerous internal and external variables” (22), in contrast to the clinical/categorical model. Hence, insight is here considered a dynamic mental state that varies over time and in intensity, depending upon internal [in the self (61)] and external (in the environment) changes (22, 40, 62). Accordingly, insight corresponds to the patient’s awareness of changes in the self that relate to his/her own pathological state and how these changes affect his/her perceptions and interactions with the world (40). Consequently, the phenomenon of insight, observed for a patient *p* at time *t* of the clinical evaluation, only reflects a given aspect of the concept of insight (37, 62).

According to the continuous model, insight is composed of three main dimensions: (1) psychical insight, which entails three sub-dimensions, i.e., clinical, cognitive and metacognitive; (2) somaesthetic insight; and (3) emotional insight (24, 30, 37, 40, 62–65) (for definitions, see **Box 1**).

Each dimension can be specifically altered. It depends upon the clinical features intrinsic to each mental illness and upon the variations of the clinical state over the illness time course. For a good insight, all these dimensions need to be preserved. This positively impacts prognosis, adherence to treatment, acceptance of hospitalization, and risks of relapse.

### Lack of Insight Relates to the Clinical State and Is Not Specific to Psychoses

The clinical model has prevailed in last decades. Consequently, lack of insight has long been considered a clinical marker distinguishing psychotic from neurotic disorders, namely, a stable and specific symptom of schizophrenia. However, the use of multidimensional evaluation scales based upon continuous methodologies has led to reconsider the clinical model. Three main observations may be retained. Firstly, insight is impaired in psychiatric disorders other than psychoses. Secondly, the dimensions of insight are differentially altered according to the diseases. Thirdly, insight varies over time and in intensity depending upon the clinical state. This is also observed in schizophrenia.

#### Deficits in Insight in Different Psychiatric Disorders

As an example, in a sample of 421 schizophrenic patients, 32.1% were found to have a low insight, 25.3% a fair insight, and 40.7% a good insight [(5); see also (66)]. In a sample of 431 patients with OCD, 25% had a low insight and 5% were unaware of the absurdity of their obsessions (9). These data invalidated the hypotheses of the clinical model that insight is constitutively impaired in schizophrenia but preserved in other psychiatric disorders. Lack of insight was also reported in patients with alcohol use disorders (AUD). In a sample of 452 AUD patients, nearly 75% had poor insight into their alcohol-related problems (67). This lack of cognitive insight was reflected in a significant contrast between the patients’ evaluation of their own cognitive performance, which was under-estimated, and the standardized neuropsychological evaluation by clinicians [(68) (N = 86); (69) (N = 91); see also (70, 71)]. Similarly, AUD patients evaluated their apathy and executive functions deficits as significantly less severe (N = 38), compared to their family members’ ratings (N = 38) (72). Yen et al. (67) further reported that a greater metacognitive insight is associated with a more accurate self-estimation of the addictive symptomatology, i.e., with a greater clinical insight (N = 425).

Collectively, these studies heightened the hypothesis by Lewis (18) that insight is not useful to distinguish psychosis from neurosis insofar as lack of insight and good insight may be equally observed in both kinds of disorders [see also (22)]. According to Marková and Berrios’s criticism (23), the empirical investigation of insight has been mostly limited to psychosis for two main reasons. Firstly, it is due to that “loss [of insight] is so dramatically apparent” (23) in psychoses, compared to neuroses. Secondly, it is due to an insufficient analysis of the concept of insight. It has led to ignore the multidimensional nature of insight and its variations over the illness time course. In general, this suggested that the quantitative analysis of insight should not prevail over its qualitative analysis.

#### Insight Correlates With the Clinical State

In addition, insight has been reported to correlate with the clinical state. It is worth noting that this correlation size is often small [see (73)]. As an example, a meta-analysis on 30 studies showed that insight negatively correlates with the severity of symptoms (global, positive, negative) in schizophrenia (74). Lack of insight was found to predict rehospitalization after 18 months following schizophrenic decompensation [(75); (N = 33); see (73)]. The severity of insight deficits at admission is also a significant predictive marker of symptoms worsening after 12 months [(76); (N = 278); see (73)].

Importantly, insight changes over the illness time course. This is the case in OCD [(9); (N = 431)]. But also in AUD, in which insight depends upon abstinence span, probably reflecting a decrease of the neurotoxic effects of alcohol on the activity of the frontal and temporo-parietal cortices [(77); (N = 117)]. This was also reported in first-episode schizophrenia: the worsening of insight deficits over the first 6 months after the disease onset predicts later functional impairments or relapse [(78); (N = 131)]. Early improvement of insight is, thus, considered an important marker for positive clinical outcome. In a sample of 670 stabilized patients with schizophrenia or schizoaffective disorders, the improvement of insight over 1 year was associated with an amelioration of the symptoms severity (79). This was also observed in a cohort of 614 schizophrenic patients with long-acting risperidone (80).

To sum up, the status of insight, in psychiatric disorders including schizophrenia, is generally associated with the patient’s clinical and symptoms-related state and varies over time and in intensity. Accordingly, insight resembles more a variable state than a stable trait.

### The Contribution of the Default Mode Network to Insight

Lack of insight is likely associated with functional abnormalities in the default mode network (DMN) (81, 82). The DMN, which is a largely distributed brain network (**Box 3**), sustains thoughts, imagination, memory and cognitive processes that relate to the Self (81, 82). Accordingly, it is considered a system underpinning internal, self-referential, affective, and introspective processes. The DMN neuro-functional feature is that its activity increases during state of unconstrained cognition (at rest).

Box 3 **Brain systems involved in insight and empathy.**

**Default Mode Network (DMN):** The DMN is thought to sustain insight. It encompasses the posterior cingulate cortex, restrosplenial cortex, ventro- and dorsomedian prefrontal cortex, precuneus, inferior parietal lobule expanding to the temporo-parietal junction, and hippocampal formation.

References: (81, 83–94)

**Fronto-Parietal Network (FPN):** The FPN is anti-correlated with the DMN. It encompasses the lateral frontal and parietal regions, anterior insula, and medial frontal cortex.

References: (94–96)

**Mirror Neuron System (MNS):** The MNS is thought to sustain automatic embodiment.

Isomorphic activations have been found in the motor system—i.e., in the inferior frontal gyrus, inferior parietal lobule, premotor cortex and superior temporal sulcus when individuals empathize with the other’s *motor action*. Similar functional isomorphism has been also reported in the anterior part of the bilateral insula, rostral part of the anterior cingulate cortex, cerebellum, and brainstem in *emotional empathy*. This holds also true for the secondary somatosensory cortex when people empathize with the *somatosensory experience* of someone else.

References: (36, 97–106)

**Vestibular System:** It plays an important role in balance, perceptions of one’s own-body and motion, location of one’s own-body in space, and, spatial navigation. It is also involved in empathy, enabling individuals to mentally locate themselves in the other’s body position in space. It encompasses the temporo-parietal junction, superior temporal gyrus, inferior parietal lobule (angular/supramarginal gyrus), posterior insular cortex, hippocampal formation, somatosensory cortex, precuneus, cingulate gyrus, and frontal cortex (motor cortex and frontal eye fields).

References: (35, 36, 107–109)

**Theory-of-Mind Network (ToM-Network):** The higher-order cognitive processes of empathy generate activations in the ToM-Network. That is, in the ventro-dorsomedian prefrontal cortex, temporo-parietal junction, anterior part of the superior temporal sulcus, precuneus, and temporal poles. Especially, the ventromedian prefrontal cortex and left temporo-parietal junction respectively code for the other’s psychological perspective and perspective ownership.

References: (36, 110–114)

**Executive System:** The right dorsolateral prefrontal cortex within the executive system sustains self-regulatory and inhibitory processes. These enable to decouple between self- and other-centered computational mechanisms in empathy..

References: (33, 36, 112, 113, 115, 116)

**Limbic System:** It plays an important role in emotional/affective and mnemonic processing when individuals are empathizing. It encompasses the thalamus, hypothalamus, basal ganglia, hippocampus and amygdala. The hippocampus and amygdala are specifically involved in empathy, respectively sustaining reactivations of information from one’s own past and embodiment and affective processing.

References: (117–122)

The DMN together works with the fronto-parietal network (FPN) (**Box 3**). In contrast to the DMN, the FPN is “task-positive”. Its activity increases during externally-focused attention and cognition (94). At rest, the DMN activity increases whereas the FPN activity decreases. Conversely, during externally-focused cognition, the FPN top-down modulates the DMN activity, enabling to disengage from internal and self-referential processing (95, 96).

In addition to neurological diseases (e.g., epilepsy and dementia), DMN dysfunctions have been observed in psychiatric disorders, such as schizophrenia (83, 87, 90), autism (91, 92), deficit/hyperactivity disorder (84, 88), OCD (93, 94), anxiety (86), and depression (85). For instance, the DMN activity and DMN-FPN functional connectivity during external stimuli-based tasks and at rest are abnormal in OCD patients [(93); (N = 18)]. These brain data are coherent with the phenomenology of the disease. The disruption between the processing of ongoing internal thoughts and external information is considered to reflect a lack of clinical and cognitive insight in OCD [(94); N = 30]. In schizophrenia, ACC activations within the DMN negatively correlate with the severity of symptoms while lower metabolism in the PCC tends to negatively correlate with the severity of insight deficits [(123); (N = 20)].

### The Complexity of Insight Growth: Developing an Objective Perspective on One’s Subjective Pathological Experiences

The complexity of insight is observable at three levels. Firstly, insight is multidimensional. Secondly, it varies over time and correlates with the patients’ clinical state. Thirdly, insight growth necessitates the patients’ capacity to develop an *objective* perspective on their *subjective* pathological experiences (18, 124). That is, a process in which the Self is an object of reflexion (26)—or a “self-as-object” processing (125). David designated it as the ability “to see ourselves as the others see us” [(21); see also (19, 20)]. It refers to the ability “to reflect upon the self’s inner world from the imagined perspective of the other” (26).

For that, insight growth (**Box 1**) has been posited to involve both self-reflexion (126) (i.e., metacognition) (127) and cognitive perspective-taking capacities (i.e., social cognition) (18–21, 26). These respectively enable to focus on oneself and to shift perspective (18–21, 26).

#### Insight Growth Combines Intact Self-Reflection and Cognitive Perspective-Taking Capacities

##### Self-Reflexion and Metacognition

Self-reflexion is considered a core feature of metacognition which is broadly defined as “cognition about cognition” (127) or “thinking about thinking” (128). Self-reflexion enables to reflect on thoughts, intentions, emotions, beliefs, etc. as these specifically depend upon oneself, i.e., as one’s owns (129) [see also (45)]. It further enables to adopt a critical viewpoint on one’s own opinions and interpretations and to reconsider them according to different potential perspectives on the same situation and event [(130); see (126)] (**Box 2**).

At the clinical level, self-reflexion reinforces “the patients’ capacity and willingness to observe their mental productions and to consider alternative explanations” (30). Accordingly, self-reflexion capacities are thought to facilitate insight. Conversely, lack of insight is considered being partially due to impaired self-reflexion capacities (21, 130–132).

##### Cognitive Perspective-Taking and Social Cognition

Cognitive perspective-taking as used in ToM is a core feature of social cognition (133, 134). Enabling to shift perspective and understand the psychological perspective of other people (135–137), it is considered necessary for insight growth in addition to self-reflexion (18–21, 26) (**Box 2**). In 1PP perspective-taking, individuals understand the other’s mental state on the basis of simulation processes (138) (**Box 2**) and “from the inside looking out” (26). In 3PP perspective-taking, individuals understand the other’s mental state on the basis of logical inferences (135) and “from the outside looking in” (26). 1PP- and 3PP-PT are fundamental for appropriate social cognition and successful self-other interaction.

Only a few studies investigated the association between lack of insight and impaired cognitive perspective-taking. Moreover, these focused on schizophrenia. Drake and Lewis (47) failed to report a relationship between difficulties in 3PP-PT ToM tasks and lower insight (N = 33). In contrast, other studies showed that 3PP-PT ToM deficits negatively impact insight [(27) (N = 58); (26) (N = 30); (29) (N = 58)]. This effect was independent of neurocognitive impairments and symptoms severity (26, 29). In the study by Bora et al. (27), ~30% of the variance in patients’ insight scores was explained by their performance to second-order ToM tests (assessing the capacity to understand what the protagonist of a story thinks about the thoughts of a second character) (26). Langdon and Ward (26) found that only deficits in ToM tasks with indirect instructions (neither directly evoking mental state inferences nor directly prompting to recruit inferential processing) correlated with lower insight. This was not the case in ToM tasks with direct instructions [directly asking what *X* thinks about (…)].

#### Summary and Questions

To sum up, theoretical hypotheses and empirical data (18–21, 26, 30, 37, 124, 130, 132) suggest that insight growth relies upon the relationship between intact self-reflexion and cognitive perspective-taking capacities.

As pointed out by Langdon and Ward (26), it is worth noting that there is lack of insight (1) when the patient’s perspective on his/her subjective pathological experiences is inaccurate and not conformed to the reality and (2) when the patient is at the same time unable to adopt the objective perspective that other individuals have on him/herself. However, the patient does not need to adopt the perspective of others on him/herself when his/her own perspective on his/her pathological experiences is already accurate. It means that self-reflexion and cognitive perspective-taking capacities are needed to improve insight when it is altered, i.e., for insight growth. In this case, it is only the combination of these two capacities that enables the patients to objectively look at themselves and their pathology from the outside, as if from the others’ viewpoint. It results in what we have lately termed the “process of objectification of oneself” (37). That is, a process in which the self and its pathological experiences—or “the self as a diseased subject”—is a consistent object of thought. That is, a process in which the patient is objectively observing himself/herself from the perspective of someone else.

However, there are still important issues that need to be overcome. Firstly, how can it be explained that indirect ToM tasks better predict insight in patients with schizophrenia, compared to direct ToM tasks (26)? Secondly, how can it be explained that certain psychiatric patients (including schizophrenics) recognize the symptoms of their disease in others (46), have ToM task performances than fall within the normal range, but are, at the very same time, unaware of their own symptoms (see 26)? Thirdly, how can be it explained that schizophrenic (30) and OCD patients (31) recognize their mental illness but do not accept it?

We here argue that the relationship between self-reflexion and cognitive perspective-taking capacities are not sufficient for insight growth. These are sufficient for the recognition of the illness but not for its acceptance. We hypothesize that insight growth rather relies upon the association of undamaged self-reflexion and empathic capacities.

Before describing our conceptual model in more details in the third section (see, *The Contribution of Self-Reflexion and Empathy to Insight Growth in Psychiatric Disorders: Proposal for a New Conceptual Framework*), we come back below to the phenomenological and neuro-functional features of empathy.

## Phenomenology of Empathy

Empathy is the capacity to feel and understand the lived experiences of someone else while mentally adopting his/her visuo-spatial and psychological perspective and maintaining self-other distinction (34, 36, 116, 139, 140). Phenomenological analyses [e.g., (141–146)] and neuroimaging research [(e.g., 33, 34, 36, 120, 137, 147, 148)] importantly contributed to understand the complex and multifaceted nature of empathy.

### The Multidimensional Approach of Empathy

Over the two last decades, behavioral and brain data evidenced that the association of perspective-taking and feeling is the hallmark of empathy. These have also shown that the empathic perspective-taking has specific features that are not encountered in other closely related but nevertheless distinct socio-cognitive functions, such as first- and third-person perspective-taking ToM (34–36, 149, 150). Indeed, empathy specifically relies upon body-related mental imagery and transformation.

#### Spatial Decentering in Empathy: The Role of Mental Body Transformations

The English neologism “empathy” has been coined by E. B. Titchener in 1909 as a translation of the German term “Einfühlung” that was introduced by R. Vischer in 1872^1^. “Einfühlung” means literally “to feel [*fühlen*] into [*ein*]” someone else (36, 145, 156, 157). This “feeling” enables to accede to the embodied mind of others “in their bodily and behavioral expressions” (146). It corresponds to the experience of one’s physiological, bodily, and affective states and changes (159) that are internally generated by the perception of another individual’s lived experience (motor, somatosensory, emotional, intentional etc.). the prefix “ein” refers to a process of mental decentering of oneself into the other.

##### Heterocentered Visuo-Spatial Perspective-Taking in Empathy

The prerequisite for empathy is to be “[… ] aware that you are outside and have to reach inside the other one” (156). Spatial decentering (**Box 2**) (160) is first required to mentally locate oneself into someone else (161). This mental location into the other corresponds to a visuo-spatial perspective-taking process (**Box 2**). It relies upon specific body-related mental imagery and transformations.

That is, individuals imagine their own-body to be located in the other’s body position. They mentally experience the world from this heterocentered [centered on the other’s body; (162)] reference frame (34, 116, 149). Hence, empathy modulates two key phenomenological components of so-called bodily self-consciousness (163): self-location [the experience of where I am in space; (164)], and egocentered perspective [the experience from where I perceive the world; (164)] (149).

##### Preservation of the Self-Other Distinction

However, self-other distinction needs to be maintained simultaneously when empathizing. It enables individuals to appropriately feel and understand that the observed experiences are originally not their own lived experiences but those of someone else. For that, the awareness of being located at a specific position in space within one’s bodily borders (egocentered reference frame) is concurrently required although it is partially top-down controlled (33, 36). Accordingly, empathy relies upon a dynamic interplay between egocentered and heterocentered visuo-spatial mechanisms. That is: (1) a mental shift from an egocentered to a heterocentered reference frame, enabling to imagine oneself in the other’s body position. And (2) a parallel although top-down regulated coding of one’s body position in space, maintaining self-other distinction at a minimal level and ensuring a balanced self-other relation (36, 116). This dynamic interplay enables to feel what the other “as other” is feeling, i.e., as the other is precisely not me.

#### The Automatic Embodiment, Heterocentered Visuo-Spatial Perspective-Taking, Emotional/Affective, Cognitive, and Self-Regulatory Processes of Empathy

In addition to heterocentered visuo-spatial perspective-taking, empathy encompasses automatic embodiment processes (enabling to internally reproduce what another person is experiencing) [e.g., (105, 159, 165)], emotional/affective processes (103), cognitive ToM-like processes (enabling to represent the other’s mental state) [e.g., (113, 136, 137, 166, 167)], and self-regulation processes (maintaining self-other distinction at the emotional, visuo-spatial, and cognitive level) (33, 36, 112, 113, 115, 116, 168) (see **Box 2**).

The embodiment and emotional/affective processes correspond to the “feeling” features of empathy. The heterocentered coding and ToM-like processes correspond respectively to its spatial and cognitive components.

### The Neuro-Functional Networks of Empathy

The complex and multifaceted nature of empathy is reflected at the neuro-functional level. This is observed in the integration of parallel but also competing activations in the Mirror Neuron System (MNS), Theory-of-Mind Network (ToM-Network), Executive, Limbic, and Vestibular Systems (**Box 3**).

#### Functional Integration of the Mirror Neuron System, Theory-of-Mind Network, Executive System, and Limbic System in Empathy

MNS and ToM-Network have long been considered mutually exclusive (169). However, functional magnetic resonance imaging (fMRI) studies lately demonstrated parallel activations and functional integration in the MNS, ToM-Network, executive system, and limbic system when empathizing (117–121). As an example, judging changes in the affective states of a cartoon protagonist who is suffering from ostracism triggers in the observers co-activations in the anterior part of the ToM-network (superior temporal sulcus (STS) and ventromedian prefrontal cortex (vmPFC)) and in the limbic regions (amygdala and hippocampus).

This co-recruitment of top-down neocortical and bottom-up limbic components suggests that empathizing with the affective states of someone else relies upon the use of cognitive representations related to the other’s mental state (vmPFC), embodiment and affective processing (amygdala), and reactivation of information from one’s own past experiences (hippocampus) (120). Moreover, empathizing with fictional characters who experience moral dilemma and difficult emotional decision-making generates a bidirectional functional connectivity between areas in the MNS, ToM-Network, and limbic system (122).

#### Contribution of the Vestibular System to Visuo-Spatial Perspective-Taking in Empathy

##### Recruitment of the Insula, Right, and Left Temporo-Parietal Junction in Empathy

Using electrical neuroimaging (EEG), we showed that the brain vestibular system (**Box 3**) significantly contributes to empathy (35). It was found to sustain the shift from the egocentered to heterocentered visuo-spatial perspective. This was reflected in activations in the left insula and right TPJ at ~60–330 ms post-stimulus onset (PSO) and in the bilateral TPJ but predominantly in the left hemisphere at ~520–630 ms PSO (35, 36).

##### The Temporo-Parietal Cortex and the Bodily Self-Consciousness

The right TPJ encodes self-location, egocentered perspective and bodily self-consciousness under normal conditions (170, 171). This is in line with data from neurological or psychiatric patients with anatomical lesions or dysfunctions in the TPJ and experiencing pathological forms of self-location (107, 108, 172–178). In out-body experience (OBE), patients experience to be located outside their own-body borders at an elevated position in space and see their physical body from this perspective (179). In heautoscopy (HAS), patients see a reduplication of their own-body in extracorporeal space, facing them, with a “preservation of the lateral asymmetries” (180). They have further difficulties in deciding whether they are located within their own-body borders (egocentered reference frame) or within the autoscopic body (heterocentered reference frame) (179). HAS is considered the pathological pendant of the interplay between egocentered and heterocentered visuo-spatial mechanisms in empathy (155, 180). Most often, OBE and HAS are respectively associated with abnormalities in the right and left TPJ (164, 181).

Accordingly, this sequence of activations in our data, i.e., firstly in the left insula and right TPJ and, secondly, in the bilateral TPJ, confirmed that individuals, when empathizing, shift from the egocentered to heterocentered visuo-spatial perspective. This supports the hypothesis that visuo-spatial perspective-taking based upon mental own-body transformations is a key empathic process. Moreover, the later co-activation of the right and left TPJ suggests that individuals computed *at minima* their own egocentered perspective in parallel with the coding of the other’s visuo-spatial perspective. This co-activation enables to maintain a basal reference to oneself and, thus, self-other distinction (35).

#### Differential Modulation of the Mirror Neuron System, Vestibular System, Executive System, and Tom-Network in Empathy

Using cortical dynamics and neural generators analyses, we reported that empathy, in addition to the vestibular system, also generates parallel activations in the MNS, ToM-Network, and executive system. Moreover, these parallel activations were differentially modulated, depending upon the time course of mental processes, i.e., embodiment, visuo-spatial perspective-taking, self-regulation, and cognitive processing (36). Activations in the MNS progressed from the right STS to the right inferior frontal gyrus (IFG) *via* the middle temporal gyrus (MTG) and inferior parietal lobule (IPL) between ~60 and ~420 ms post-stimulus onset (PSO). The vestibular system was recruited at ~60–630 ms PSO. Here, activations progressed from the insula and right TPJ (~60–330 ms) to the bilateral TPJ (~520–630 ms). The right dlPFC was specifically activated at ~330–420 ms. Finally, activations within the ToM-Network were found in the STS, right TPJ, temporal poles (~60–330 ms), left TPJ, and precuneus (~520–630 ms) between ~60 and ~630 ms.

Hence, our data confirm that empathy relies upon the integration of parallel activations in cooperating and/or competing brain networks, reflecting the recruitment of distinct but related mental processes. These further suggest that embodiment processes (STS, MTG, IPL, IFG), occur in parallel with (1) shifting from the egocentered (insula, right TPJ) to heterocentered visuo-spatial perspective (left TPJ), (2) decoupling computational mechanisms between self- and other-centered processes (right dlPFC), and (3) higher-order cognitive representations (temporal poles, left TPJ, precuneus). We did not report activations in the vmPFC. This is probably due to that our experimental paradigm and tasks did not involve complex judgments about the others’ mental states. Another possible explanation is that vmPFC activations occur later in the neural time course. We focused on a time window from 0 to 700 ms PSO as the quality of the EEG signal was poorer after this time period (due to stimulus-locked analyses). Thus, it is probable that vmPFC occurred after 700 ms. The precise time window of the vmPFC activation in empathy needs to be tested in future EEG study.

### Concluding Remarks: The Subjective and Objective Dimensions of Empathy

Collectively, neuroimaging studies converge on the contribution of cooperating and/or competing parallel brain networks to empathy, confirming its multifaceted and complex nature. EEG studies further shed light on the activations time course within these brain networks. Importantly, this neural time course informs on how visuo-spatial perspective-taking operates in empathy. Indeed, it enables to disengage oneself from one’s own present experience, thoughts, beliefs, feelings, and emotions, etc. that are early encoded in the egocentered reference frame (insula/right TPJ). It is done in order to mentally locate one’s own-body into the body position of another individual and to internally experience his/her experiences, thoughts, beliefs, feelings, and emotions from his/her body position (heterocentered) (bilateral/left TPJ) (to feel *into*).

Embodiment (MNS) and emotional/affective processes (anterior part of the ACC, brainstem/dorsal pons, cerebellum) also occur simultaneously. These correspond to the physiological, bodily, and affective states and changes (159) that are triggered in individuals when they are internally experiencing the others’ experience (*to feel* into). This feeling results from sharing and embodying the other’s mental state. It corresponds to the subjective dimension of empathy.

Moreover, visuo-spatial perspective-taking with the help of cognitive ToM-like (temporal pole, precuneus, vmPFC) and self-regulatory (dlPFC) processes enables to comprehend as objectively as possible the others’ experience. That is, what the other *as other* is experiencing. This corresponds to the objective dimension of empathy.

Finally, the parallel reference to oneself that is encoded in the right TPJ and insula enables to compare what the other is experiencing with one’s own current experience. It enables to adapt one’s own behaviors toward the other and to correct one’s first assessments and predictions concerning the other’s mental state. It further maintains self-other distinction^2^.

## The Contribution of Self-Reflexion and Empathy to Insight Growth in Psychiatric Disorders: Proposal for a New Conceptual Model

There are empirical and clinical arguments in favor of the contribution of self-reflexion and cognitive perspective-taking capacities to insight growth. However, two important issues need to be addressed.

### Limitations and Theoretical Hypotheses

#### Two Related Issues

Firstly, as pointed out by Langdon and Ward (26), difficulties in indirect ToM tasks in patients with schizophrenia have a more negative impact on insight, compared to difficulties in direct ToM tasks. It suggests that performances to tasks in which patients compute more spontaneous, implicit, or automatic processing better predict insight than tasks in which a more explicit and controlled processing is calculated [for comparable results, see (150); for a criticism, see (182)]. These data are in line with the simulation theory account of ToM, positing that ToM relies upon 1PP-PT capacities. Therefore, Langdon and Ward (26) concluded that theoretically reasoning about the other’s thought (3PP-PT) (which is tapped by direct ToM-tasks) without simulating his/her perspective (1PP-PT) (which is tapped by indirect ToM-tasks) would not be sufficient for insight growth.

Secondly, as above-mentioned, clinical studies reported that certain psychiatric patients with schizophrenia (30) and OCD (31) are able to recognize their mental illness but are not convinced of this. That is, patients do not accept their disease.

Is this latter observation inconsistent with the hypothesis that impaired 1PP-PT ToM capacities negatively impact insight? Does it suggest that these are only sufficient for the recognition of the mental illness but not for its acceptance? Accordingly, 1PP-PT ToM capacities would explain psychical insight (recognition) but not emotional insight (acceptance).

#### Hypotheses

To overcome these limitations, we propose a new conceptual model. It aims to explain the dysfunctional mechanisms underpinning lack of insight in psychiatric disorders. We here further deepen our first theoretical approach published elsewhere (37).

##### Main Working Hypothesis

Our main working hypothesis is that insight growth relies upon the relationship between intact self-reflexion and empathic capacities. Accordingly, we suggest that 1PP-PT ToM capacities, even associated with intact self-reflexion, are not sufficient for the development of insight. The starting point of our working hypothesis is the assumption by Langdon and Ward (26) that deficits in 1PP-PT but not 3PP-PT ToM have a negative impact on insight. This is a very important and suitable hypothesis. However, we here aim to partially modulate this approach and bring further precisions.

There is no doubt that 1PP simulation ToM and empathy are two close phenomena. However, they distinguish for two related reasons. Firstly, empathy entails embodiment and affective processes (*to feel* into) whereas 1PP simulation ToM does not. It means that empathy is not only a cognitive and mental simulation of the other’s mental state. It is also a way to embody and to affectively process this mental state. In empathy, the feeling stems from both these embodiment and affective processing (159). Thus, empathy does not only consist in “imagining what it would be like to be in the “mental shoes” of another person” (26) as in 1PP simulation ToM but also in internally experiencing what he/she is experiencing.

Secondly, according to simulation theoreticians, individuals, when simulating, use and mentally project onto someone else their own perceptive, emotional, and cognitive schemas and patterns (183–185). This potentially leads to egocentric biases (186). In contrast, individuals, when empathizing, inhibit the tendency to project their own schemas and patterns onto the other. It enables to feel and to understand what the other *as other* is experiencing. That is, precisely as the other *is not me*. For instance, this inhibition of projective processes in association with a basal self-other distinction (see second section *Phenomenology of Empathy*) is fundamental to helping behaviors and medical care (116, 157, 158).

Thus, we argue that feeling into another individual enables to internally and more efficiently experience his/her mental state than do 1PP simulation ToM processes. As a consequence, this subjective experience (i.e., based on feelings), in association with basal self-other distinction and inhibition of projective processes, would enable to understand more objectively the other’s mental state and to facilitate the affective adherence to the other’s thoughts.

But how feeling into someone else may positively impact insight and its two stages, i.e., the recognition and the acceptance of the mental illness? Our main working hypothesis further divides into two sub-hypotheses.

##### First Sub-Hypothesis: The Process of Objectification

Firstly, we hypothesize that intact heterocentered perspective-taking, embodiment, and cognitive processes, in association with self-reflexion, are necessary and sufficient for the recognition of the mental illness. It is not the case for its acceptance. That is, (a) looking at oneself and one’s pathological mental experiences from the visuo-spatial perspective of another individual (heterocentered), (b) embodying his/her current thought about oneself, and (c) understanding this thought as being specifically related to oneself result in what we have previously termed a “process of objectification of oneself” (37).

This process of objectification enables to recognize one’s own illness (“I am suffering from *this* mental illness”). It generates an objective viewpoint on the self. This would, thus, selectively impact psychical insight, particularly metacognitive insight and clinical insight (**Box 1**).

##### Second Sub-Hypothesis: The Process of Subjectification

Secondly, individuals need to have recognized their mental illness before being able to accept it. However, if the process of objectification enables to recognize one’s own mental illness, it is not sufficient to accept it. Additional mechanisms probably occur.

Indeed, “to have the capacity to adopt another’s point of view as so to imagine what it would be like to think something different than one actually believes about self” is completely different from “to be able to accept that the other person’s point of view provides the more accurate representation of the true state of affairs” (26). Accordingly, the other’s point of view needs not only to be objectively understood but also incorporated into the subjective representation of the self. We hypothesize that, if the mental illness has been already recognized (process of objectification), then, the association of both intact self-reflexion and empathic affective capacities contribute to accept the illness (“This mental illness is *mine*”). It means that the affective experience of the other’s thought about oneself enables to subjectively adhere to this thought. Hence, it facilitates acceptance.

It has been well-documented that higher-order cognitive processes, such as acceptance or decision-making for example, require computational processing by the cognitive system but also the adhesion of the emotional system to the cognitive evaluation (187). “Any behaviour is by definition both cognitive and affective” (188). Brain data confirmed that emotion and cognition are “non-modular” (188) as these are integrated in specific areas, such as the lateral PFC (189, 190), orbito-frontal cortex, vmPFC and ACC (188).

We argue that affectively experiencing the thought of another person about oneself reinforces the adherence of the emotional system to the objective evaluation and recognition of oneself “as a diseased subject” (process of objectification). It refers to what we here propose to label a “process of subjectification”. This would, thus, selectively impact emotional insight (**Box 1**).

##### Summary

To sum up, we posit that the process of objectification positively impacts psychical insight, leading to recognize one’s mental illness. The process of subjectification positively impacts emotional insight, leading to accept one’s mental illness. The process of objectification needs to be intact in order that the process of subjectification occurs. That is, there is no emotional insight if the psychical insight is impaired. In other words, the mental illness must have been already recognized to be, then, accepted. Insight as a whole process is still impaired—although less severely—if the recognition of the illness is intact (psychical insight) but its acceptance is altered (emotional insight).

### Applications and Predictions

What are the applications and predictions of our conceptual model? We insist on that our model does not only focus on schizophrenia but aims to apply to psychiatric diseases in general. Firstly, as evidenced by the continuous model and clinical data, insight is not a symptom of the disease but a mental state that varies over the illness time course and in intensity. Secondly, lack of insight has been reported in different psychiatric conditions. Hence, lack of insight is not specific to schizophrenia. Thirdly, deficits in empathic capacities are also observed in most psychiatric diseases [for a review, see (115)].

Therefore, there are solid clinical and empirical arguments to examine the relationship between lack of insight and deficits in empathy in psychiatric disorders. Moreover, and in accordance with our hypothesis, cognitive and affective empathy has been shown to enhance insight in schizophrenia (32). We posit that the negative effect of the association of impaired self-reflexion and empathic capacities on insight is a transnosographic state in psychiatric diseases. We further argue that endophenotypical differences modulate the expression of this common state, determining a given disease as specific.

Below, we focus on schizophrenia, OCD, and BD.

#### Transnosographic State and Endophenotypical Modulations

We here note that, on the basis of prior studies, the data from at least 100 patients for each clinical group are needed to verify our conceptual model (i.e., to get a sufficient statistical power).

##### Schizophrenia

Firstly, in accordance with prior studies (78, 79), we predict that during acute and decompensation episodes, there is a complete breakdown of both self-reflexion and empathic capacities, with a deleterious effect on insight. In stabilized patients, self-reflexion and empathic capacities may be differentially altered and, thus, the dimensions of insight differentially impaired. This would depend upon symptoms features.

###### Schizophrenic Patients With Preeminent Negative Symptoms

Patients with a prevailing negative symptomatology are more impaired in emotions recognition, general social abilities, ToM performance, and empathy (150, 191–193), especially when their symptoms resemble those of autism (194, 195), in comparison to patients with a prevailing positive symptomatology. In a previous work (150), we showed that patients with negative symptoms were unable to spontaneously empathize with others. That is, they were impaired in inhibiting their egocentered visuo-spatial perspective and disengaging from themselves. We here predict that patients with a predominant negative symptomatology lack psychical insight because of deficits in heterocentered visuo-spatial perspective-taking capacities (hypo-functionality). Thus, the process of objectification is here impaired. Consequently, patients do not recognize their own illness.

###### Schizophrenic Patients With Preeminent Positive Symptoms

In contrast, we found that patients with prevailing positive symptoms have augmented facilities in using heterocentered visuo-spatial coding and imagining themselves in the body position of others (150). This is line with a study by Thakkar and Park (196), reporting that facilities in inhibiting one’s egocentered perspective positively correlate with increased positive syndrome schizoptypy. This is further concordant with the hypothesis by Abu-Akel (197, 198) that patients with a predominant positive symptomatology have exaggerated ToM competences. These aberrant capacities would be responsible for the patients’ tendency to overrate and over-attribute intentions, thoughts, emotions, etc. to other individuals, as in delusions (191, 197, 199).

We predict that patients with positive symptoms have normal empathic heterocentered visuo-spatial perspective-taking capacities. They are able to imagine themselves in the others’ body position. However, they fail to appropriately feel, i.e., embody, what others are feeling. This yields to misinterpretations and misattributions at a higher-order cognitive level. Accordingly, we anticipate that deficits in empathic embodiment processes (hypo-functionality) trigger compensatory exaggerated empathic higher-order cognitive processes (hyper-functionality). The process of objectification is altered, negatively impacting psychical insight. There is no recognition of the mental illness.

In both clinical groups, lack of insight and associated alteration of empathic processes would vary over the illness time course and in intensity, depending upon the severity of symptoms and clinical state.

###### Intact Recognition but Altered Acceptance of the Mental Illness in Schizophrenic Patients With Either Negative or Positive Symptoms

Certain schizophrenic patients with a relative good insight recognize “the logical assertion that they are suffering from a mental illness” (15) but they are still not convinced of this (30). We posit that the process of recognition is here intact due to the preservation of the heterocentered, embodiment, and cognitive processes of empathy. This positively impacts the psychical insight. In contrast, the affective process of empathy is altered. It means that patients are unable to affectively experience the other’s thought about themselves and, thus, to subjectively adhere to this thought. This negatively impacts emotional insight. We predict that this insight status is observed in patients with either positive or negative preeminent symptoms when the clinical state is significantly improved.

##### Bipolar Disorder

Manic episodes are well-documented to be associated with lower insight (200, 201). Recent data demonstrated that depressive episodes also correlate with deficits in insight, although the correlation size was smaller than for manic episodes (202). Deficits in insight during depressive episodes were especially associated with higher rates of suicide ideation. Moreover, empirical studies using the Interpersonal Reactive Index [IRI; (139)], i.e., a self-report questionnaire evaluating empathy, or the Multifaceted Empathy Test [MET; (203)] reported that manic and depressive episodes are associated with deficits in the cognitive processes of empathy (204–206). In contrast, manic BD patients had higher affective empathy than depressives and controls (204–206). This effect was interpreted as reflecting excessive affective empathic reactions due to disturbances in emotion inhibition and persistence of positive emotions (207). This way of perceiving social stimuli as more positive than these really are leads patients to the conviction to fully understand other individuals (206).

###### BD Patients in Manic Episodes

We predict that BD patients in manic episodes have intact empathic heterocentered visuo-spatial perspective-taking and are, thus, able to imagine themselves in the body position of others. However, they have altered empathic cognitive processes (hypo-functionality) and exaggerated embodiment and affective processes (hyper-functionality). There is a parallel alteration of the process of objectification and process of subjectification, negatively impacting psychical and emotional insight. That is, patients feel positively the negative content of the other’s thought about themselves (e.g., the clinical evaluation of their physician), leading to misinterpretations.

###### BD Patients in Depressive Episode

In contrast, we predict that BD patients in depressive episodes have difficulties in inhibiting their own egocentered visuo-spatial perspective and using a heterocentered visuo-spatial coding (hypo-functionality). They are, thus, unable to disengage from themselves. In this case, the process of objectification is altered, leading to deficits in metacognitive and clinical insight. This triggers an augmentation of negative thoughts related to oneself and ruminations, potentially leading to suicide. We hypothesize that a comparable process may be also observed in unipolar depression.

##### Obsessive-Compulsive Disorders

Foa et al. (9) reported that, 70% of a sample of 431 OCD patients had a good insight, 25% a low insight, and 5% were unaware of the absurdity of their obsessions (see section *Phenomenology of Insight*).

###### OCD Patients With No Insight

We here predict that OCD patients with no insight have high difficulties in disengaging from themselves and using a heterocentered visuo-spatial perspective (hypo-functionality). This negatively impacts psychical insight, and, thus, the recognition of the mental illness. This would be more associated with egocentered OCD, i.e., a sub-clinical group in which patients suffer from obsessional fears concerning themselves (e.g., “I will contract a fatal illness, if I do not clean the house”), or OCD with impulsion phobia.

###### OCD Patients With Low Insight

In contrast, we predict that OCD patients with a low insight have preserved empathic heterocentered, embodiment, and cognitive processes (208). The process of objectification is here intact. However, the affective process of empathy is altered (hypo-functionality). Patients are unable to affectively experience the other’s thought about themselves and, as a consequence, to subjectively adhere to this thought. This would lead to an alteration of the process of subjectification, impacting emotional insight and acceptance of the illness. That is, patients recognize the absurdity of their obsessions but are not convinced of this. Here, this would be encountered in patients whose “(…) affective states thus seem to take precedence over their rational thoughts” (31).

#### Model Limitations and Future Research Protocols

##### Limitations

There are potential limitations to our conceptual model. Clinical and neuro-functional hypotheses posit that psychiatric disorders may be explained by dysfunctional cognitive flexibility, associated with functional abnormalities in the prefrontal and frontal cortices (209–211). If correct, this would suggest that difficulties in developing an objective viewpoint on one’s subjective pathological experiences are not triggered by impaired self-reflexion and empathic capacities but by control and executive dysfunctions. A recent meta-analysis based on brain data from 5,728 controls and 5,493 psychiatric patients with schizophrenia, bipolar or unipolar depression, anxiety and SUDs reported transdiagnostically abnormal activations in the left PFC, anterior insula, left vmPFC, right intraparietal sulcus, and mid-cingulate/pre-supplementary motor area (212). This suggests that psychiatric disorders rely upon a common pattern of cognitive disruption. These brain areas further overlap with the DMN, likely sustaining insight, and empathic brain networks. Hence, the precise contribution of cognitive flexibility to insight needs to be further examined.

As described in the first section, the clinical model considers insight a symptom of the disease whereas the continuous model considers insight a variable mental state. Clinical and empirical data seem to validate the continuous model. The same question needs to be addressed to lack of empathy. As an example, diminished empathic capacities seem to pertain to the symptom structure in OCD with impulsion phobia. Conversely, exaggerated empathic capacities seem to be a symptom of hetero-centered OCD, i.e., which is characterized by obsessional and inappropriate fears concerning other individuals and associated rituals and compulsions. Hence, our model should disentangle whether deficits in empathy are a symptom of the disease or a variable mental state, depending upon the symptoms severity and clinical state.

Our model also needs to take into account the negative effect of insight on depressive comorbidity. For instance, it is well-documented that insight improves in schizophrenic patients when these are stabilized. However, this improvement of insight is associated with an increase of the comorbid depressive symptoms. It is probably due to that patients become aware of the gravity of their disease. Hence, the effect of a good—or better—insight on depressive comorbid symptoms must be explored. Another issue is also to understand how depressive comorbidity may negatively impact, in turn, empathic capacities.

##### Research Protocols

Only a very few studies investigated the relationship between lack of insight and deficits in empathy. It means that our model is rather speculative and needs to be further tested in clinical, empirical, and neuroimaging studies. For that, it is necessary to develop extensive research protocols with different psychiatric groups and at different phases of the clinical state (first-episode onset, decompensation, stabilization, or remission). These protocols should entail: (1) clinical evaluation targeting symptoms severity [as an example in case of OCD: The Yale-Brown Obsessive-Compulsive Scale (Y-BOC); (213)] and insight status [Brow Assessment of Beliefs Scale (BABS); (214)]; (2) self-evaluation of symptoms severity [Obsessive Compulsive Inventory (OCIR), (215)], insight [Birchwood Insight Scale (IS); (216)], and empathy (IRI); (3) standardized neuropsychological test batteries; (4) measure of brain activity at rest (using fMRI or EEG); and (5) batteries of behavioral paradigms that evaluate insight [e.g., adaptation of the Insight Task by (217)] and empathic processes (hetero-centered visuo-spatial perspective-taking capacities (E.S.T; 149); ToM-processes [“Joke Appreciation Task”; (218)]; emotional processing [“Reading the Mind in the Eyes Test; (219); MET] using fMRI or EEG. Clinical, behavioral, and neuroimaging studies based on extensive research protocols with different psychiatric populations (schizophrenia, OCD, and AU) are in progress in our laboratory.

## Conclusion

In the present contribution, we proposed a new conceptual model aiming to explain the dysfunctional mechanisms underpinning lack of insight in psychiatric disorders. We posited that the association between impaired self-reflexion and empathic capacities negatively impact insight. Moreover, we distinguished between two new concepts: the process of objectification and the process of subjectification. We showed that the process of objectification results from empathic heterocentered, embodiment, and cognitive processes. Generating an objective viewpoint on oneself, it enables to recognize one’s own mental illness and, thus, positively impacts psychical insight. We further showed that the process of subjectification results from empathic affective processes. We argued that affectively experiencing the thought of another person about oneself reinforces the adhesion of the emotional system to the objective evaluation and recognition of the illness. Hence, the process of subjectification enables to accept one’s mental illness and positively impacts emotional insight.

Furthermore, applying our conceptual model to different psychiatric conditions, we predicted that the negative effect of impaired self-reflexion and empathic capacities on insight is a transnosographic state. Moreover, we predicted that endophenotypical differences modulate this common state. Hyper vs. hypo-functional empathic processes (heterocentered visuo-spatial perspective, embodiment process, or affective processing, etc.) would impact differently psychical or emotional insight depending upon symptoms features and clinical state in each psychiatric disease.

Although based upon prior clinical, behavioral, and neuroimaging studies, our model is rather speculative. If validated by empirical data, our model would be helpful to develop new cognitive-behavioral therapies and neuro-stimulation protocols adapted to each psychiatric disease in each clinical phase (first onset, acute episode, stabilization, remission). This would be fundamental to ameliorate the quality of care.

## Author Contributions

BT and NJ theorized the link between lack of insight and deficits in empathy in psychiatric disorders. BT theorized the process of objectification, process of subjectification and predictions of the conceptual model. GH-G and NL respectively helped with the elaboration of the clinical and neuro-functional hypotheses. BT wrote the manuscript.

## Funding

This work was promoted by the “Unité de Recherche Clinique intersectorielle en psychiatrie à vocation régionale du Centre Hospitaliser Henri Laborit (Poitiers, France)” which received a financial support from the “Agence de Santé Régionale en Nouvelle Aquitaine”.

## Conflict of Interest

The authors declare that the research was conducted in the absence of any commercial or financial relationships that could be construed as a potential conflict of interest.

## Footnotes

1 R.Vischer firstly introduced the term “Einfühlung” in his thesis manuscript *Über das optische Formgefühl* in 1872 and further developed the concept of “Einfühlung” in two later articles (*Der ästhetische Akt und die reine Form* in 1874 and *Über ästhetische Naturbetrachtung* in 1890 (151)). He used this term to refer to the human capacity to “feel [fühlen] into [ein]” in the field of aesthetics (arts and nature). In 1913, T.Lipps applied the term “Einfühlung” to the field of psychology (152). E.B. Titchener coined the english neologism “empathy” as a translation of “Einfühlung”, on the basis of the Greek term “empatheia”. The question whether “empathy” appropriately reflects the original meaning of “Einfühlung” is still a debated issue [see, (153, 154); for a detailed etymological analysis, see 145; see also (155)]. We here refer to empathy as “to feel into” on the basis of the German term as in 36, 116, 150 [for a comparable approach, see as (34, 156–158)].

2 We here note that empathy distinguishes from sympathy in that it maintains self-other distinction whereas sympathy does not [see (36, 144)].
